# Development of methods to identify digitally excluded older people, and tailoring of interventions to meet their digital needs: a protocol for a mixed-methods study (the INCLUDE study)

**DOI:** 10.1136/bmjopen-2025-102723

**Published:** 2025-09-18

**Authors:** Caroline Brundle, Jessica Faye Johansson, Kate Best, Andrew Clegg, Anne Forster, Thomas Atkinson, Marilyn Foster, Sara Humphrey, Alison Iliff, Jessica Inglis, Claire Walker, Liz Graham

**Affiliations:** 1 Academic Unit for Ageing and Stroke Research, Bradford Teaching Hospitals NHS Foundation Trust, Bradford, UK; 2 Academic Unit for Ageing and Stroke Research, University of Leeds, Leeds, UK; 3 PPI Co-applicant, Hull, UK; 4 Bradford District and Craven Health and Care Partnership, Bradford, UK; 5 North East and Yorkshire Region Office for Health Improvement and Disparities, UK Department of Health and Social Care, London, UK; 6 Age UK Leeds, Leeds, UK

**Keywords:** Digital Technology, Aging, Frail Elderly, Primary Health Care, Internet

## Abstract

**Abstract:**

**Introduction:**

Digital inclusion (which includes skills, accessibility and connectivity to the internet and digital devices) is a ‘super social determinant of health’ because it affects many aspects of life that influence health. Older people are especially vulnerable to digital exclusion. Existing digital inclusion interventions are commonly offered opportunistically to people who come into contact with services, or in specific locations. The lack of systematic identification of need unintentionally excludes older people who may be most in need of support, and that support is not addressing their needs.

**Methods and analysis:**

This multi-method project includes six workstreams: (1) A survey of people aged 65+ to ask about digital use and engagement. Survey data will be used to develop a model that predicts digital exclusion from data available in primary care records. (2) Testing, via a further survey, the external validity of the model to identify those who are digitally excluded. (3) Interviews with community service providers to identify, understand and define the components of existing digital inclusion services for older people. Concurrently, a rapid review of the literature will identify evidence for interventions aimed at supporting digitally excluded adults aged 65+. (4) Interviews with people aged 65+ representing a range of digital use will explore factors from the COM-B model that influence digital behaviours—their capability (C), opportunity (O) and motivation (M) relating to digital engagement. Analysis outputs will identify the intersectional nature of barriers or facilitators to digital inclusion. (5) Co-production workshops with older people and community service providers will identify key components of interventions that are required to address digital exclusion. Components will be mapped against existing interventions, and the ‘best fit’ intervention(s) refined. An implementation plan will be developed in parallel. (6) Feasibility testing of the refined intervention(s) to assess acceptability and obtain feedback on content and delivery mechanisms.

**Ethics and dissemination:**

This study was approved by the Yorkshire & The Humber - Bradford Leeds Research Ethics Committee on 23 October 2023 (ref. 23/YH/0234). Findings will be disseminated in academic journals and shared at webinars, seminars, conferences and events arranged by organisations operating across the digital inclusion and older people fields.

**Trial registration:**

https://www.isrctn.com/ISRCTN18306736

**STRENGTHS AND LIMITATIONS OF THIS STUDY**

- The study team are working with general practitioner (GP) practices that include some of the least and most deprived populations in the UK, inclusive of a range of minority ethnic groups, thus ensuring that findings will have relevance across the UK population.
- A key output of the work will be a model, using routine GP data, to support identification of older people who are digitally excluded. The methodology developed may be appropriate for replication for identification of other hardly-reached groups.
- Qualitative interviews and workshops will be focused on an in-depth understanding of digital exclusion, and associated behaviours and factors that influence digital behaviours, to provide a comprehensive understanding of what the intervention(s) we develop should target.
- The study team’s strong links with GP practices, local authorities and the community are facilitating recruitment and engagement and will provide direct routes for dissemination.
- While we endeavour to maximise diversity of inclusion in our study population, we are working in one region of the UK (Yorkshire) so there may be variations in the population in other areas of the UK that we do not capture.

## Introduction

 Digital exclusion is a super social determinant of health^1 2^ that affects a number of different areas of life that influence our health and well-being. These areas include: finance (online banking, benefits guidance); physical and social environment (online tickets for trains, parking, cultural events); health and social care (access to e-consultations and remote/online healthcare, online appointment booking systems, internet-based information about health, social care and housing); and online interaction with family members who do not live locally. There is a widening gap between those who can and cannot (or do not) use technology, which was hastened by the increased use of technology in our everyday activities during the COVID-19 pandemic. While there is no universally recognised definition of digital exclusion,^3^ the term typically describes those who do not have access to or capability to use the internet and are missing out by not being online,^4^ or whose limited online activity or digital skills mean they do not fully participate in modern society.^3 5^

Digital exclusion has the most negative impact on older people (eg, it can contribute to loneliness, poor housing and poor health); however, older people are the group most often excluded from digital activities,^6^,^9^ with 31% of over 65s not having an internet connection at home, compared with 4% among those aged 35–44.^3^ Among older people, digital exclusion is reportedly higher in lone households, those of Asian ethnicity and females, with these inequalities appearing to increase with age.^10 11^ Income and socioeconomic disadvantage have also been associated with digital exclusion, although this has not been examined by age.

Numerous reports (eg,^12^,^19^) have highlighted the extent and challenges of digital exclusion, but prevalence data has been derived from national surveys, which have inherent weaknesses (eg, they are often not granular enough to provide detail on the nuances of people’s lives),^20^ and there is no current, systematic method to identify those who are digitally excluded^21^ at a local population level. More focused work to identify barriers and facilitators to digital inclusion has overwhelmingly relied on convenience sampling,^20^ usually people already in receipt of services or self-identifying as requiring support. These approaches do not reliably identify the ‘hardly reached’ older person who may be most in need.

Third sector organisations have developed digital inclusion interventions to address the needs of their local populations, but access to this digital support is often reliant on third party or self-referrals, or geographical location as a ‘best guess’ of the digitally excluded population,^22^ while evidence of success is often anecdotal.^20^ Thus, there is likely to be a ‘hidden’ population of older people who are digitally excluded^7^ who are not known to support services and therefore support available is not tailored to their needs. The evidence review from the Digital Poverty Alliance^20^ identifies that ‘comparatively little academic evidence on the digital divide comes from qualitative studies, lived experience or co-produced research with people who experience digital poverty in the UK’, and highlights the need for community level data. In line with their recommendations, we will develop a systematic identification process; explore through qualitative interviews the determinants and intersectional nature of digital exclusion; gain insight and understanding from third sector groups; and co-produce with the hardly reached digitally excluded population tailored interventions to address their needs.

## Methods and analysis

### Study aims and objectives

The overarching aim of this research is to develop a robust, replicable identification system for digitally excluded older adults; and, working with community groups and those who are digitally excluded, refine and feasibility test an intervention (or interventions) to improve digital inclusion opportunities for those currently excluded.

Study objectives are to:

A. Identify those who are digitally excluded:

1. Survey methods will be used to estimate prevalence and predictors of digital exclusion in those aged 65+. Using this survey data, we will then use a predictive modelling approach that is implementable in primary care systems to systematically identify older people (aged 65+) at risk of digital exclusion or who are digitally excluded.
2. Feasibility test our method of identifying those who are digitally excluded from primary healthcare registers.

B. Refine an intervention to meet their needs:

1. Identify the content, format and purpose of existing digital inclusion interventions through regional community consultation, and undertake a rapid review of the literature to understand the emerging evidence for digital training/support interventions for older people.
2. Explore through interviews with a sample of older people the determinants and intersectional nature of digital exclusion.
3. Undertake a series of co-production workshops—involving digitally included and excluded older people, and service providers—to identify and develop key components of an intervention(s) that would engage those who are currently digitally excluded (considering also how the intervention would be implemented in practice).

C. Feasibility test the intervention:

1. Provision of the newly-developed intervention to digitally excluded older people—to ascertain inclusion, feasibility, acceptability and inform intervention refinement (its content and how it is provided).

### Design summary

**Figure 1:**
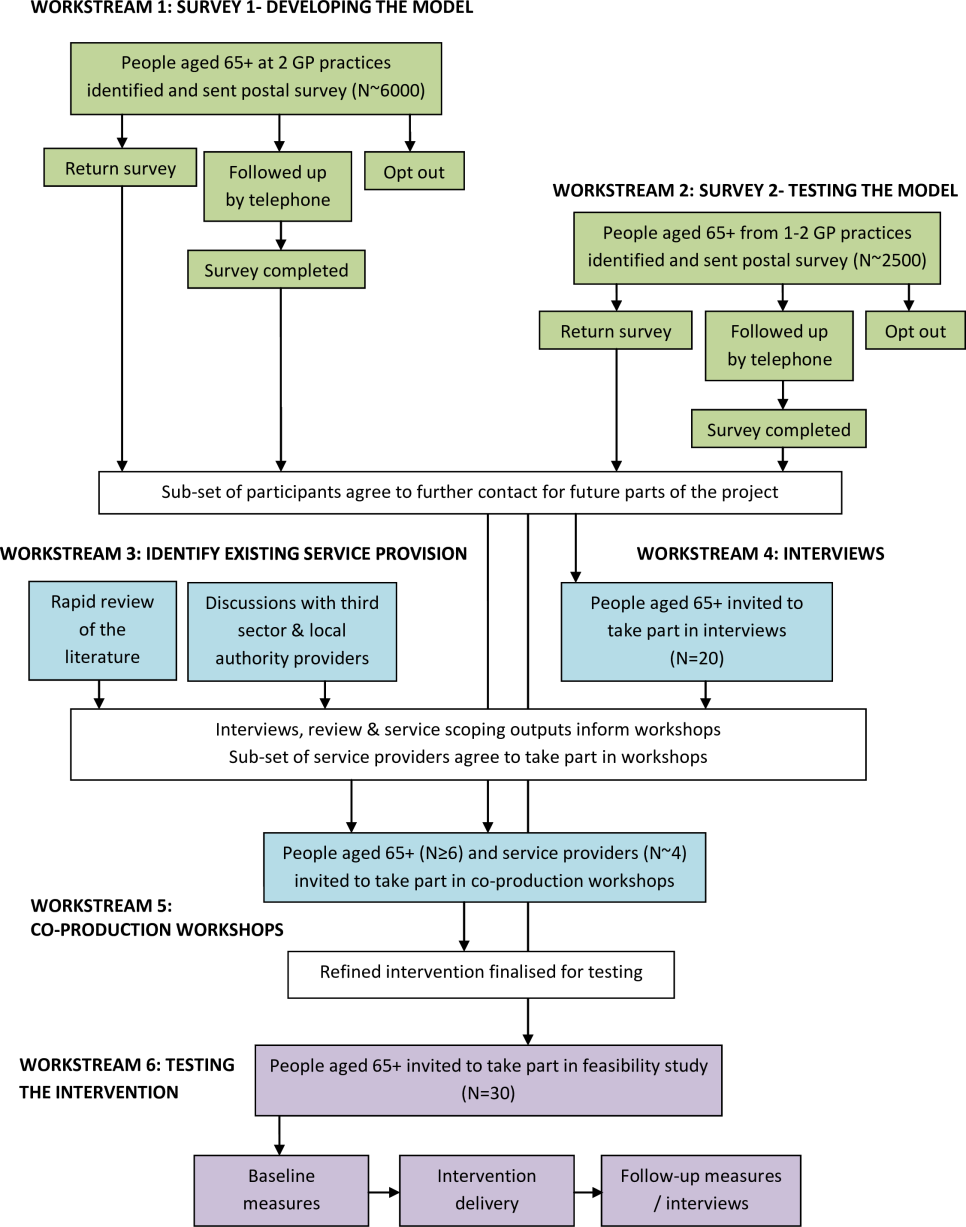
Study flowchart. GP, general practitioner.

This multi-method study, taking a pragmatic world view^23^ and having consideration for the Medical Research Council guidance for developing and evaluating complex interventions,^24^ comprises six workstreams which align with the above objectives. See figure 1 for a summary of these workstreams.

### Setting

Two general practitioner (GP) practices will be selected to take part in the first survey, and a further one to two practices in the second survey (workstreams 1 and 2). The number of practices selected to be determined by numbers of older people on practice registers and the required sample size. Primary care health registers provide the most inclusive population record available, so GP practices are the most suitable setting for survey distribution. We will work with practices that include some of the least and most socioeconomically disadvantaged populations in the UK, those with varying age profiles, and will ensure that our sample includes people from a range of minority ethnic backgrounds and from both urban and rural areas.

Scoping work with community service providers (workstream 3) will mostly take place remotely via telephone or video call, possibly with some visits to the organisations. Interviews with older people (workstream 4) will take place in participants’ homes or another community location of their choice. Co-production workshops (workstream 5) will be held in a community venue, accessible to participants. While we cannot specify the setting for the feasibility study to test the newly refined intervention (workstream 6) until it is developed, we expect it to be delivered in a suitable community venue. In all cases, participants will be given the option to engage with research activities over the telephone or a video platform if they have concerns about meeting a researcher or group face-to-face, or if they are unable to travel to a central venue.

### Eligibility (older people)

Surveys (workstreams 1 and 2) will be sent to patients at participating GP practices who are aged 65+, excluding those who have opted out of data sharing for research purposes, or who are on end-of-life care pathways. Participants approached to complete the surveys via community groups and through researcher face-to-face contact will be eligible if they are aged 65+ and are willing and able to take part.

Survey respondents who indicate their willingness to be contacted about further research activities on returning their surveys will be eligible to take part in one of: interviews (workstream 4); co-production workshops (workstream 5); or the feasibility study (workstream 6). However, we will exclude from these workstreams those who:

- lack capacity to take part even with the help of a supporter, or
- have no consultee available or willing to support involvement where they lack capacity to consent.

Language is not an exclusion criterion: where participants do not speak English, we will endeavour to provide translator support for interviews and workshops; alternatively, the support of a relative or friend who can help with translation will be sought.

### Eligibility (service providers)

Those providing digital training or support to older adults and who are independent of the research team will be invited to take part in our service scoping work (workstream 3) and the co-production workshops (workstream 5).

### Identification of digitally excluded older people

#### Workstream 1: survey

##### Design

A bespoke survey will incorporate elements necessary for modelling purposes. It will include relevant and appropriate internet usage questions from the Office for National Statistics annual survey (last use of the internet, frequency of use, use of internet for specific tasks),^25^ and known and potential predictors of digital exclusion (age, gender, partial postcode, living status (alone/with others), ethnicity).^36^,^9 26 27^ Layout and presentation of the survey will be informed by best practice (colour of paper, font size, etc) and we will be mindful of literacy levels and incorporate pictorial elements to maximise engagement. Patient and public involvement and engagement (PPIE) will support survey design, content and approach.

##### Outreach work

Prior to sending the surveys, we will identify older people’s community groups that fall within the catchment areas of participating GP practices, visiting them to explain the research and provide flyers for group attendees. Groups might include those organised by charities such as Age UK, informal community groups (eg, a lunch club, U3A), religious communities such as those attending a mosque or those at day centres or sheltered housing. This outreach work is intended to facilitate attendees’ engagement with surveys received later via their GP, as well as foster relationships for future in-person recruitment approaches.

##### Recruitment

A member of the clinical team at participating GP practices will identify eligible patients in their registers by running a prespecified search provided by the research team. Also included in the output of eligible patients will be a unique study ID, contact details, electronic Frailty Index (eFI) score (the eFI uses existing information held in electronic health records to measure frailty based on the accumulation of a range of deficits)^28^ and data items such as age, gender and postcode. These data will enable targeted telephone follow-up of non-responders to increase the representativeness of our sample.

GP practices will send via post to each eligible patient an introductory letter explaining what the study is about, followed 1 week later by: an invitation to participate, a participant information sheet (PIS), a survey and a ‘keeping in touch’ form on which participants can, optionally, provide their contact details if they wish to take part in later workstreams. They will also be sent a prepaid envelope for survey return directly to the research team. DocMail, a recognised secure method for contacting large numbers of patients for clinical purposes, will be used to minimise workload for GP practices, while retaining patient confidentiality. Those who do not wish to take part in the study will have a 2-week window following survey receipt to opt out from survey completion and any further study contact by returning the blank survey or telephoning the research team. Thereafter, we will augment our recruitment approach by a researcher based at each GP practice telephoning non-responders (an approach made clear in the PIS) to ask if they would be willing to complete the survey over the telephone. It will not be possible to follow-up all non-responders due to the volume of surveys sent, so the follow-up of participants will be prioritised using factors known to predict digital exclusion (eg, older age, female gender, socioeconomic deprivation derived from postcode, frailty).

Surveys will also be distributed by researchers directly to older people at community groups, clinics, day centres and sheltered housing—those engaged during the earlier outreach work—as well as via other community contacts across our recruitment catchment area including areas with varying levels of deprivation. Researchers will offer to administer surveys directly with community group members who are in attendance when they visit, offering support with survey completion (eg, providing language support, or reading questions for those with visual impairment). Researchers might also visit participating GP practices and geriatric medicine outpatient clinics to invite attending patients to complete the survey.

##### Data management and statistical analysis

GP practices will provide a list of study IDs with associated eFI scores to incorporate into the modelling work—an agreed approach enabling the research team to identify frailty status for survey respondents without obtaining any identifiable patient data. Index of Multiple Deprivation (IMD) will be derived from partial postcode (eg, AB99 1) entered by participants in the survey. We will compare demographic information of responders (eg, age, sex, eFI score) to that of the whole population of patients to whom surveys were sent. This whole population data will be obtained in summary form from the list of eligible participants generated at the practices.

A ‘hard’ definition of digital exclusion (ie,total non-use) will be used in the survey data analysis. Thus, participants will be defined as digitally included if they have independently (without help) performed one or more of the 22 online tasks specified in our survey, and digitally excluded if they have not performed any of these tasks. The tasks fall into four broad categories of online use: entertainment, healthcare, communication and buying things/managing money. However, we also wish to consider those who are narrow users and, as such, disadvantaged by not being able to fully engage with online activities.^10 18 26^ We will therefore also define participants as wide or narrow users, where wide users are those who perform one or more tasks in each of the four categories, and narrow users are those who do not perform at least one task in each category of use.

Summary statistics will be produced to describe prevalence of digital exclusion and narrow use. Logistic regression will be performed with digital exclusion as the binary outcome, with candidate predictors of digital exclusion being age, gender, IMD, ethnicity, frailty category and lone living status. Using a ‘multilevel analysis of individual heterogeneity and discriminatory accuracy’ (MAIHDA) approach,^29^ participants will be assigned to strata based on categories of the above predictors. For example, a strata might be white women aged 65–75 with mild frailty, living alone in an area with high levels of socioeconomic deprivation. The variance will be compared between the model with and without the strata random effect, which indicates whether there are interaction effects (known as intersectionality) between the variables. Using the MAIHDA, precision weighted estimates of digital exclusion can be produced for each strata, with the raw predictions being further weighted towards the fixed effect estimates as the strata size gets smaller. Should there be no evidence of interactions, we will revert to a fixed effects logistic regression model which would enable us to include different functional forms of the included predictors (eg, age as a continuous rather than categorical predictor), and to consider including other predictors (such as memory and sight problems). As a sensitivity analysis, we will also refit the model with narrow/wide use as the binary outcome. If sensitivity analysis shows similar characteristics of those who are non-users and narrow users, both would be included in our definition of digitally excluded used in later workstreams.

Data will be coded and analysed using statistical software Stata. The model will be sense-checked with participants during the interviews and workshops in workstreams 4 and 5, respectively.

##### Sample size

With a conservative response rate of 15%, there would be 900 responses from a mail-out to 6000 people. Assuming digital exclusion affects 31% of respondents^3^ and with a conservative R^2^ of 0.1, we will have adequate power to perform fixed effects logistic regression with 11 predictor parameters.^30^ For the MAIHDA model, selection and categorisation of the variables will be driven based on the sample size of participants in each category and strata, with an aim of≥10 in all strata and>1 expected event (ie, digital exclusion) per strata.^29 31^

We expect a response rate of>15% and R^2^>0.1; however, our other recruitment strategies will augment the sample size, should response rates from mail-outs be lower than expected.

### Workstream 2: feasibility testing the identification approach

#### Design

We will perform an external validation of the model developed in workstream 1. Methods described for workstream 1 will be used to design and distribute the survey from GP practices, with content refined to include the appropriate predictive factors included in our model.

#### Sampling

All patients at the participating GP practices will be screened for eligibility. We will work with different GP practices to those involved in workstream 1 to ensure that we do not target the same patients who have already taken part.

Fewer participants are required for external validation^32 33^: we require a minimum sample of 200 cases (100 who are digitally excluded and 100 digitally included). Working with one to two GP practices with a population pool of>2500 aged 65+ should ensure sufficient responses are received. However, the response rate observed during workstream 1 will inform our target population pool.

We will select GP practices that serve a large and diverse population of people aged 65 and over, systematically approaching those meeting eligibility criteria. In addition to the systematic sending of surveys, as for workstream 1, we will also purposively sample non-respondents to increase the representativeness of our sample.

#### Recruitment

Sample identification and consent will proceed as described for workstream 1; however, recruitment materials and approach will be adapted following review of workstream 1 responses and considering the developed model.

#### Data management and analysis

We will apply the model developed in workstream 1 to predict which older people are digitally excluded. Using the survey responses, we will compare these predictions to the reported digital exclusion status of participants. Predictive performance statistics will be used to produce optimism-adjusted estimates for calibration (eg, calibration-in-the-large, observed/expected), discrimination (eg, area under the curve) and overall (eg, Nagelkerke R^2^) performance of predicted risks.

### Refine an intervention to meet the needs of digitally excluded older people

#### Workstream 3: mapping existing service provision

##### Literature review

A rapid review of the literature^34^ will identify existing evidence for interventions and approaches aimed at reducing digital exclusion of adults aged 65+. Informed by existing reviews (eg,^714 15 18^,^20^) a search strategy will be developed, focused on the most recent 10 years, and run in key social science/multidisciplinary databases: Dimensions, Scopus and Web of Science. Grey literature will also be searched. Studies will be included that: are written in English, include adults aged 65+ and focus on interventions or approaches to address digital exclusion.

Intervention delivery mechanism, content and ‘dose’, assessment of compliance and measures of effectiveness will be extracted from included articles. When extracting data from eligible articles, the COM-B model^35^ and the Theoretical Domains Framework (TDF)^36^ will be used to establish which domains current interventions target. Data will be narratively summarised and will contribute to the case scenarios (personas) developed in workstream 4 that will be the basis for the co-production workshop discussions held during workstream 5.

##### Defining existing local services

Organisations providing digital training and support services for older people will be identified through: our collaborating community partners (Age UK Leeds, Carers’ Resource Shipley), internet searches, snowball sampling and discussions with colleagues in the Integrated Care Boards. A member of staff from organisations that are willing to take part will be invited to have a short discussion with a researcher. They will be asked to articulate the target population, format, content and purpose of their current offer. This information will be recorded and mapped by the research team, and cross-checked with the providers to ensure accuracy and understanding. Researchers will also explore the availability of free or discounted digital devices, and available benefits or charitable services which might support digital access. Community centres or locations where people can use devices, or charge and use their own will be identified.

When defining existing local services, we will also apply the COM-B and TDF to understand the purpose of current interventions. As with the rapid review of the literature, these outputs will contribute to the case scenarios that will be the basis for the co-production workshop discussions (workstream 5).

### Workstream 4: interviews with older people to explore determinants of digital exclusion

#### Design

Semi-structured interviews will be undertaken to build on responses provided in workstream 1 and will enable an in-depth understanding of digital exclusion, associated factors that influence digital behaviours, and individuals’ experiences. The COM-B model^35^ will be used to explore the complexities of digital exclusion and will provide a structure for understanding and organising the determinants of behaviours into capabilities (C), opportunities (O) and motivations (M). Michie *et al* (2014) proposed an individual must have the capabilities, opportunity and motivation, for a given behaviour to occur. Behaviours can also influence these three components, and capabilities and opportunity can influence motivation. Using this model will facilitate a comprehensive understanding of what the intervention(s) should target before moving towards solutions. Interview findings will inform co-production processes (workstream 5).

#### Sampling

Approximately 20 participants who agreed to further contact when they returned their workstream 1 surveys will be interviewed. We will sample participants purposively, based on characteristics that have been identified in the literature as influencing digital exclusion, for example, age, gender, living status, deprivation, ethnicity, as well as a range of digital use (eg, regular user, limited user, non-user) as specified in their survey responses. To ensure maximum variation in these factors, we will sample a range of ages from the following categories: 65–74, 75–84 and 85+, equal amounts of males and females, people in different living circumstances (alone or with others), people from postcodes with varying deprivation indices, and different ethnicities which will be selected to provide a representative sample based on the proportions identified across participating GP practices. We will select participants based on these factors until variation saturation is reached. The intention of this approach is to ensure our results reflect the diversity of needs and experiences of people aged 65 years and over.

#### Recruitment

Potential participants will be telephoned by a researcher to establish interest. Where the telephone conversation identifies concerns with capacity, the researcher will ask to speak to someone else who might support the person’s involvement, if feasible. If no one can be identified to support the person, and the researcher feels that they do not have sufficient capacity, participation will not be pursued. All those who are eligible and show an interest in participation will be sent a study information sheet by post or email. Written consent will be obtained for those with capacity, while agreement to participate will be provided by an identified personal consultee (a relative, friend or carer) on behalf of a participant who lacks capacity to consent for themself.^37^

An inclusive approach to interviews will be adopted wherever possible: we will suggest that participants (regardless of capacity) may invite a carer or friend to support them if they wish—for example, if they have hearing loss or visual impairment. Identified supporters will receive information and provide consent in the same way as participants.

#### Data collection

A researcher will interview each participant using a topic guide which will include: extent of digital use (eg, internet and device use); what internet/devices are used for (eg, finances, socialising); whether technology helps or hinders management of health and social aspects of life; the extent to which they feel included/excluded and the associated impacts; financial concerns; privacy and security concerns; digital needs and how interventions can better address their needs and skills; and factors that influence digital behaviours.

Steps will be taken to adapt the interview methods to be inclusive, for instance by adapting language used, using explanatory images and writing down key words for people with communication or cognitive difficulties. With their permission, a record of participants’ profiles will be kept so we are able to report on the inclusiveness of our sample.

#### Data analysis

Researchers will use a thematic approach to analysis^38^: data familiarisation, followed by open coding each transcript to describe units of meaning. Units of meaning will be compared and contrasted to produce thematic categories. Cross-case analysis will compare the categories within each transcript and produce key themes across the data. The COM-B model^35^ will be used as a sensitising construct. When working through the inductive analysis process, we will consider the components of the COM-B model and the subdivisions of the components when we are developing the themes (eg, psychological and physical capability; social and physical opportunity; and automatic and reflective motivation). We will also explore intersectionality to capture the complexities that might explain why some individuals may be more or less digitally excluded. The fundamental assumption of intersectionality is that factors contributing to the diversity of experiences such as age, gender and ethnicity are not unidimensional.^39^ Considering how these factors may be interlinked will help to inform why people have different experiences and a subsequent need for different support. Our analysis approach ensures that the findings are still grounded in the data but also organised in a way that will be informative for the later co-production process (workstream 5). Personas (case scenarios) will be developed from the data to communicate findings in the workshops. Summary statistics will be produced to describe the included sample.

### Workstream 5: co-production workshops to refine an intervention

#### Design

Co-production recognises and uses the skills and expertise of various stakeholders to develop interventions ‘with’ rather than ‘for’ them.^12 40^ A co-production approach will be employed to identify key components of an intervention (or interventions) to address digital exclusion.

Relevant domains from the TDF^41^ will be applied to the data to establish specific determinants within each of the COM-B components. For example, the domain ‘knowledge’ aligns with capabilities; ‘environmental context’ aligns with opportunity; ‘beliefs about capabilities’ aligns with motivation. Using the COM-B in combination with the TDF provides a clear theoretical understanding of what needs to be targeted in intervention(s) for digitally excluded individuals.

We will use the Behaviour Change Taxonomy^42^ to identify the behaviour change techniques (BCTs) that may be used to address the determinants of digital exclusion. For example, the intervention may aim to enhance someone’s beliefs about their capabilities to access the internet and find resources; BCTs related to self-beliefs may be selected, for example, self-talk.

#### Sampling and recruitment

At least six adults aged 65+, and approximately four representatives from organisations that provide digital training/support to older adults will be invited to take part in co-production workshops. We may also invite a health/care professional or social prescriber to take part in some or all of the workshops if it is expected that the intervention may include a health component, or they might be involved in intervention pathways.

Eligible older adults will be purposively selected from the pool of those who responded to the workstream 1 survey and agreed to be contacted. We will include older adults with a range of digital experience to ensure diversity of views. They will be recruited using the same approach outlined for workstream 4.

Digital service providers and health/care professionals will be purposively selected from our large pool of contacts established during workstreams 1 and 3, including those that provide a variety of services to a diverse range of individuals.

Where participants are unable to attend workshops, speaking to them separately at home or over the telephone to elicit their views will be explored. If more than one person drops out from workshop attendance, the recruitment of additional participants will be considered to ensure representativeness of views. Reasons for drop-out will be explored, as these may be relevant to the digital exclusion topic.

#### Data collection and intervention development

Four to five 2-hour workshops will be organised by members of the research team. Workshop content will be informed by earlier interviews, developed case scenarios and our review of existing evidence, alongside behaviour change theories.^35^ We will use pictorial representations of concepts and media clips to illustrate points for discussion. We will ask for and act on participants’ feedback between workshops to ensure the presentation style and content are tailored to their needs and preferences; and they are able to actively contribute to the process.

Taking an iterative approach, data from workshop activities and audio recordings will be used to inform tasks for subsequent workshops and ongoing intervention refinement.

Participants will agree the ‘ideal’ intervention content and the best way to deliver an intervention (or interventions) to enhance digital inclusion (a ‘gold standard’). Currently available intervention components identified during workstream 3 will be compared with workshop participants’ ‘ideal’, and required refinements to these, as well as how to deliver the intervention in practice, will be discussed and agreed. A logic model and implementation plan will be produced.

At the end of this workstream an intervention (or interventions) will have been developed for testing in workstream 6.

### Feasibility testing the intervention

#### Workstream 6: feasibility study to explore the acceptability of the intervention

##### Design

We will undertake a mixed-methods single-arm feasibility study. A non-randomised design with quantitative and qualitative outcomes is appropriate during the early stages of intervention development, where key questions regarding intervention acceptability and feasibility need to be explored.^24^

##### Sampling

The refined intervention(s) will be tested with 30 consenting participants. A sample size of 30 is appropriate for feasibility work with exploratory and qualitative outcomes—for which no formal sample size calculation is required.^43^ Participants will be selected from those who agreed to further contact in workstreams 1 and 2, and who are digitally excluded (according to a definition to be informed by workstream 1 analysis).

##### Recruitment

Characteristics of potential participants will be reviewed by the research team to identify those who are ‘digitally excluded’. Eligible participants will be contacted by a researcher—by telephone in the first instance to establish interest. Where interest is expressed, a study information sheet will be sent. An inclusive approach to recruitment, to maximise participation opportunities, will proceed as for workstreams 4 and 5.

The proportion of those approached who agree to take part in the feasibility study, and reasons for non-participation, will be monitored. Similarly, the characteristics of participants and non-participants will be documented. Participant withdrawals will be monitored to account for missing data, with timing and reason for withdrawal documented for each participant. These data will contribute to conclusions drawn about the acceptability of the intervention (and the research) to a diverse group of older people.

##### Intervention delivery and data collection

Until we have completed workstream 5, we are not able to define the intervention, and therefore are not able to specify details such as: who will deliver the intervention, its content, format and delivery mechanisms, what data will be collected, measures to be used to monitor acceptability and feasibility or follow-up time frame. Once the intervention has been defined, we will explicitly describe its components, delivery platform and training required for those delivering it, structuring the intervention description using the template for intervention description and replication (TIDieR) framework.^44^ We will also specify planned data collection tools and timelines.

Participants will be offered the newly developed intervention(s), and asked to provide baseline and follow-up measures regarding their internet use, as well as a measure of their satisfaction with the intervention. A researcher will interview a proportion of participants (engagers with the intervention and non-engagers), as well as those providing the intervention to explore acceptability and implementation fidelity. Interview topic guides will be formulated based on the COM-B model, TDF domains and BCTs outlined in our intervention logic model, and relevant constructs related to implementation (eg, those from Normalisation Process Theory (NPT)^45^). Questions may focus on the extent to which the intervention(s) have influenced beliefs about capabilities and how individuals made sense of the intervention before delivering or using it.

##### Data analysis

Summary statistics will describe changes in internet use pre-post intervention, as well as levels of satisfaction with, and adherence to the intervention. Interview data will be analysed using the thematic approach described for workstream 4. During analysis, the COM-B, TDF, BCTs and NPT will all be used to support our understanding of whether the interventions have targeted what was intended.

Quantitative and qualitative findings will identify and describe the extent to which providers and recipients of the intervention(s) deem the intervention(s) to be acceptable and will describe whether it has been possible to implement the intervention(s) in accordance with the implementation plan. Outputs will determine necessary refinements to the intervention, logic model and implementation plan.

### Study organisation and governance

The INCLUDE study is sponsored by Bradford Teaching Hospitals NHS Foundation Trust and is co-ordinated by the Academic Unit for Ageing and Stroke Research (ASR) (Bradford Teaching Hospitals NHS Foundation Trust and University of Leeds). The Project Management Group (PMG) comprises the authors and ASR research team. Independent oversight and supervision is provided by a Project Steering Group (PSG).

All data will be stored and managed in accordance with the provisions of the Data Protection Act (2018). Survey data will be entered into a bespoke study database for management and analysis. Paper data will be checked against that entered into the database to ensure accuracy. Surveys will be stored (physically and electronically) separately to any contact details provided by participants willing to be contacted about later workstreams, rendering them anonymous. Interview audio recordings will be transcribed for analysis: in the written transcripts, participants will be given pseudonyms and any identifiable information will be removed. Data in all formats will be securely stored with access restricted to the researchers undertaking the data collection and analysis.

### Patient and public involvement

The importance of this work was identified during older people’s research priority-setting work carried out by members of the PMG and was endorsed by older people consulted during the design of the study. The PMG includes a PPIE representative, who has been involved since the grant application development stage. A PPIE group has been established and members are consulted at key time points throughout the study to discuss the project plan, to obtain their opinions on and suggestions for information materials, surveys, interview topic guides and workshop content. PPIE group members are also involved in activities such as testing surveys, reviewing data collection tools and interviews to assess time and burden of participation. The PPIE group will contribute to dissemination activities, for example, by advising on content and format of reports and outputs, and suggesting local, regional and national routes to impact. We also consult community groups in diverse settings across Yorkshire, attending a wide variety of groups for older people, including those that provide digital support services. Input has been sought on the design of our surveys and information materials. In liaising with these groups, we aim to understand older people’s experiences and suggestions for improving digital engagement, and to inform our recruitment and engagement approaches.

### Ethics and dissemination

#### Ethics approval and consent to participate

This study was approved by the Yorkshire & The Humber - Bradford Leeds Research Ethics Committee (REC) on 23 October 2023 (ref. 23/YH/0234). All protocol modifications will be submitted to the Health Research Authority (HRA) and REC, via the Integrated Research Approval System (IRAS).

Researchers will seek written consent from all participants who take part in interviews, workshops and the feasibility study. Prior to this, all participants will be provided with an information sheet and will have the opportunity to ask questions. Researchers will be trained in Good Clinical Practice and the Mental Capacity Act 2005.

#### Dissemination

The PMG will draft, and the PSG will agree, a publication plan which will be monitored by both groups throughout the project. In addition to publishing findings in academic journals, during the study we will present regular reports to our community partners, and share emerging findings at webinars, seminars, conferences and events arranged by organisations operating across the digital inclusion and older people fields. Lay summaries of findings will be produced for dissemination to participants and participating GP practices for onward dissemination to their patients. To maximise the impact of the study, we will engage with and share outputs with commissioners, policymakers, voluntary sector organisations, practitioners and older people, taking advantage of the PMG’s links with integrated care boards and partnerships, and regional local authority and third sector organisations focused on the provision of care for older people.

## Discussion

Developing a systematic, inclusive approach to the identification of digitally excluded older people is crucial to enable a meaningful exploration of their digital needs. Ensuring that interventions to facilitate digital inclusion are appropriately tailored to this group will enable optimisation and targeting of scarce resources, reducing the selective nature of current provision.

Increasing digital inclusion will help reduce inequality, and provide a platform for enhancing shared care and engagement with medical and social services.^14^ There is also an economic advantage for the individual and society.^15^ Benefits for the older person are considerable in that they can: reduce social isolation through enhanced communication; increase social engagement through awareness of local events, activities and transport links; provide cognitive stimulation through access to games and puzzles; and allow greater use of smart home devices to support daily living.

Striving to make digital access and use more inclusive will ultimately lead to more older people who are in need of support having increased digital confidence, which in turn will impact positively on their independence. For example, they may be able to use smart devices to manage or monitor long-term health conditions, and digital abilities may support ageing in place. This could improve uptake of national programmes such as virtual wards, where admissions are avoided by at-home monitoring using smart devices and/or digital connectivity.

Our work will provide a simple and replicable method for identifying digitally excluded older people, and will develop intervention materials and approaches to address their identified needs. Also, we will provide a robust template methodology for engaging with hardly reached groups which will have potential benefit for service provision and future research.

The study team is well-equipped to undertake this project, with wide-ranging experience and a PPIE co-applicant involved in study design and oversight. Our strong links with GP practices, local authorities and the community will facilitate recruitment and engagement (the study commenced in November 2023 and positive community engagement has already been achieved) and will provide direct routes for dissemination. Our national profile and links will facilitate roll-out to Integrated Care Partnerships and Boards enabling them to address the needs of the hardly reached digitally excluded, thus reducing some aspects of inequality and safely enhancing opportunities and experience for many.
