# Supplementary material for: Development of methods to identify digitally excluded older people, and tailoring of interventions to meet their digital needs: a protocol for a mixed-methods study (the INCLUDE study)
Source: BMJ Open. 2025 Sep 18;15(9):e102723. doi: 10.1136/bmjopen-2025-102723 (PMC12458810; doi:10.1136/bmjopen-2025-102723)
Supplement: Supplementary file 1 [file bmjopen-2025-102723.reviewer_comments.pdf]

PEER REVIEW HISTORY

BMJ Open publishes all reviews undertaken for accepted manuscripts. Reviewers are asked to complete a checklist review form and are provided with free text boxes to elaborate on their assessment. These free text comments are reproduced below.

ARTICLE DETAILS

Title (Provisional)

Development of methods to identify digitally excluded older people, and tailoring of interventions to meet their digital needs: a protocol for a mixed-methods study (The INCLUDE study)

Authors

Brundle, Caroline; Johansson, Jessica Faye; Best, Kate; Clegg, Andrew; Forster, Anne; Atkinson, Thomas; Foster, Marilyn; Humphrey, Sara; Iliff, Alison; Inglis, Jessica; Walker, Claire; Graham, Liz

---

VERSION 1 - REVIEW

**Reviewer**	**1**
**Name**	**Tomczyk, Łukasz**
**Affiliation**	**Institute of Education, Jagiellonian University**
**Date**	**19-Jun-2025**
**COI**	**None**

Congratulations on designing an excellent study. Digital exclusion remains a global problem, even in highly developed countries such as the UK. Multi-stage studies are an excellent opportunity to develop comprehensive solutions that can minimise this problem. Overall, I have no reservations about the methodological assumptions presented. However, a few questions arise: how can the representativeness of this type of research be ensured, particularly in rural areas where there are no religious organisations, third age universities or senior clubs? What level of generalisation will the quantitative research results have? Will the tools be piloted and will there be an external expert evaluation of the tools beforehand? Another issue is the paradigm of qualitative research. In what vein will the qualitative research be conducted? And a final brief question: is this project a continuation of previous research? What was the inspiration for it? I am keeping my fingers crossed for the diagnosis and implementation of the research results. This will be a strong study – congratulations!

**Reviewer**	**2**
**Name**	**McDonnell-Naughton, Mary**

Affiliation **Department of Nursing and Health Care Athlone Institute of Technology**

Date **01-Jul-2025**

COI **None**

Summary statistics will be produced to describe prevalence of digital exclusion and narrow use.

"Logistic regression will be performed with digital exclusion as the binary outcome, with candidate predictors of digital exclusion being age, gender, IMD, ethnicity, frailty category and lone living status. Using a 'multilevel analysis of individual heterogeneity and discriminatory accuracy' (MAIHDA) approach [27] participants will be assigned to strata based on categories of the above predictors. For example, a strata might be white women aged 65-75 with mild frailty, living alone in an area with high levels of socioeconomic deprivation. The variance will be compared between the model with and without the strata random effect, which indicates whether there are interaction effects (known as intersectionality) between the variables." This is well explained and very clear.

The challenge of getting the opinion of 6000 people may prove to be a logistic nightmare, correlating and following up with the various stakeholders, do you think this is a reasonable task? 15% response rate will be low, also what do you do about the participants who do not respond is there a difference between the different cohorts.

Patient and public involvement very good. I cannot answer questions 9 and 10 as this is work that is ongoing.

VERSION 1 - AUTHOR RESPONSE

Comment	Response	Changes
Reviewer 1		
Congratulations on designing an excellent study. Digital exclusion remains a global problem, even in highly developed countries such as the UK. Multi-stage studies are an excellent opportunity to develop comprehensive solutions that can minimise this problem. Overall, I have no reservations about the methodological assumptions presented.	Thank you for taking the time to review our work and for your supportive comments.	N/A
How can the representativeness of this	We acknowledge that the study is being carried out in one region of the UK in the summary of	Page 8

type of research be ensured, particularly in rural areas where there are no religious organisations, third age universities or senior clubs?	strengths and limitations (page 3), however, we will endeavour to recruit participants from both urban and rural areas within the region. Text has been added to the setting section on page 8 to make this explicit. We aim to understand the different digital challenges faced by older people as a result of multiple factors, which would certainly include location.<br><br>We acknowledge that it might be difficult to approach those who do not respond to the survey in rural areas where there are no community groups that we might attend to invite participation; however we do plan to monitor sample characteristics and target non-responders by telephone to increase representativeness of our sample (see p.10).	
What level of generalisation will the quantitative research results have?	By sending the surveys to all eligible patients on the GP practices' registers we believe we will achieve a large representative sample, and results that will be generalisable. We do of course acknowledge the limitations relating to working in one region. (See Strengths & limitations section page 3 and additional text added to page 7 mentioned above.)	Page 7
Will the tools be piloted and will there be an external expert evaluation of the tools beforehand?	We are unclear exactly which 'tools' the reviewer is referring to; however, we can report that the survey tool was developed in conjunction with older people's community groups and study-specific PPI group members (see p.9). In addition, any tools which we select for the feasibility study will be tested with our PPIE members.<br>Text has been added to the PPIE section on page 24 for clarity.	Page 24
Another issue is the paradigm of qualitative research. In what vein will the qualitative research be conducted?	All of the qualitative research will be conducted within a pragmatic paradigm. This allows for flexibility in the methods and focuses on practical outcomes.<br><br>We will select methods for the intervention evaluation based on what is required to be able to evaluate the use of the intervention components and whether they can be implemented by providers. We have used relevant aspects of the Medical Research Council (MRC) guidance for developing and evaluating complex interventions to guide this research. We are mindful of ensuring our research aligns with the core elements outlined in the guidance considering context; developing, refining and testing programme theory; engaging stakeholders, identifying key uncertainties, refining the intervention; and considering economic factors.	Page 7 (plus onward impact through the rest of the manuscript due to inclusion of new references

	Working within this guidance provides scope for drawing upon relevant theories in the development and evaluation of interventions, therefore we will incorporate relevant theories where needed. Amendments have been made to the Design summary section (page 7).	
Is this project a continuation of previous research? What was the inspiration for it?	This is briefly mentioned in the paper (page 24).<br><br>For further information: this work has emerged from research priority setting work undertaken by members of this team as part of the Yorkshire & Humber Applied Research Collaboration Older People with Frailty theme. In discussions with groups of older people, digital exclusion was identified as a core theme running across a number of priority areas: for example, older people talked about a lack of digital skills precluding their access to information about health conditions, or communications with health professionals, as well as limiting non-medical activities such as banking. https://arc-yh.nihr.ac.uk/research/projects/research-prioritisation/	N/A
I am keeping my fingers crossed for the diagnosis and implementation of the research results. This will be a strong study – congratulations!	Thank you very much for your kind comments.	N/A
Reviewer: 2		
Summary statistics will be produced to describe prevalence of digital exclusion and narrow use. "Logistic regression will be performed with digital exclusion as the binary outcome, with candidate predictors of digital exclusion being age, gender, IMD, ethnicity, frailty category and lone living status. Using a 'multilevel analysis of individual heterogeneity and discriminatory accuracy' (MAIHDA) approach [27] participants will be assigned to strata based on categories of the above predictors. For	Thank you for taking the time to review our work and for your supportive comments.	N/A

example, a strata might be white women aged 65-75 with mild frailty, living alone in an area with high levels of socioeconomic deprivation. The variance will be compared between the model with and without the strata random effect, which indicates whether there are interaction effects (known as intersectionality) between the variables." This is well explained and very clear.		
The challenge of getting the opinion of 6000 people may prove to be a logistic nightmare, correlating and following up with the various stakeholders, do you think this is a reasonable task?	We think the reviewer might have misunderstood our intentions in relation to the survey. We will send the workstream 1 survey to 6000 people, but this is to ensure our response rate is sufficient (at least 900). We don't expect 6000 responses. Also this is a cross sectional survey, so there is no need for any further follow-up. We will have data from GP practices which will be linked by ID to all respondents (see page 11), so the correlation in this respect is not a problem.	N/A
15% response rate will be low, also what do you do about the participants who do not respond is there a difference between the different cohorts.	15% was our estimation for the lowest numbers required from a modelling perspective.<br><br>We are able to compare the non-responders to the responders in terms of age, frailty, ethnicity, gender via anonymous data provided by the practices. Text has been added to page 11 to provide further detail.<br><br>Targeted telephone follow ups will be used to maximise representativeness of our responding sample (see page 10).	Page 11
Patient and public involvement very good.	Thank you very much for your positive comment.	N/A
